# Constrictive pericarditis: 21 years' experience and review of literature

**DOI:** 10.11604/pamj.2021.38.141.22884

**Published:** 2021-02-08

**Authors:** Taamallah Karima, Ben Zaied Nesrine, Lahdhili Hatem, Ben Omrane Skander, Denguir Raouf, Chenik Selim

**Affiliations:** 1 Department of Cardiac Surgery, Military Hospital of Tunis, Tunis, Tunisia,; 2 Department of Cardiac Surgery, La Rabta Hospital, Tunis, Tunisia

**Keywords:** Constrictive pericarditis, pericardiotomy, tuberculosis, echocardiography, prognosis

## Abstract

To the best of our knowledge there are no publications about Tunisian experience in constrictive pericarditis (CP); the aim of this study was therefore to review our twenty-one years’ experience in terms of clinical and surgical outcomes and risk factors of death after pericardiectomy. An analytic bicentric and retrospective study carried out on 25 patients (20 male) with CP underwent pericardiectomy, collected over a 21-years period. The mean age was 40.46±16.74 years [7.5-72]. The commonest comorbid factor was tabagism (52%). The most common etiology was tuberculosis (n = 11, 44%). Dyspnea was the most common functional symptom (n = 21, 84%). Pericardiectomy was performed in all our patients within 2.9±3.19 months after confirmation of diagnosis. It was subtotal in 96% of cases. The commonest postoperative complications are pleural effusion (20%). Dyspnea was regressed within 1.8 months in 80% of cases and clinical signs of right heart failure within a mean duration of 1.62 months in 53% of cases. Perioperative mortality was 12% (3 deaths), late mortality was 4% (1 patient). Cardiopulmonary bypass, New York Heart Association (NYHA) over class II and right ventricular dysfunction are the prognostic factors of mortality (p = 0.001, 0.046, 0.019). Tuberculosis as etiology of CP had no impact on mortality. CP is a rare disease, with non-specific clinical signs. Pericardiectomy is effective with a significant improvement of the functional status of patients and favorable outcome at short and long term nevertheless hospital mortality is not negligible and depends on many factors.

## Introduction

Chronic constrictive pericarditis (CP) is a serious disease fortunately rare accounting for approximately 0.5 to 2% of heart disease and 0.6% of interventions in cardiovascular surgery department. It is characterized by constriction and adiastolia [1,2]. Symptomatology of CP is not specific, making diagnosis not always easy, it is dominated by right heart failure, CP is occasionally confused with hepatic cirrhosis or furthermore restrictive cardiomyopathy or endomyocardial fibrosis making necessary the use of many complementary examinations to perform the diagnosis [3,4]. Doppler echocardiography and cardiac catheterization are the cornerstone in the diagnostic work-up, but use of multimodality cardiac imaging in particular cardiac computed tomography (CT), magnetic resonance imaging (MRI) is often necessary to differentiate between CP and restrictive myocardiopathy which represent the main differential diagnosis. Medical treatment is only symptomatic to prepare the patient for surgery, which represents the only radical treatment for CP. The surgical intervention consists of a pericardiectomy. A significant improvement of symptoms was observed in the immediate operative period in some series [5]. There are no publications and data on CP in Tunisia. The aim of this study was to describe the epidemiological, clinical, paraclinical and therapeutic characteristics of CP and identify post-operative prognostic factors, as well as short-term and long-term evolution after pericardiectomy.

## Methods

An analytic, longitudinal and retrospective bicentric study, including 25 cases of chronic constrictive pericarditis, collected over a period of 21 years from November 1994 to May 2015. Included in this study all patients underwent pericardiectomy for CP. Pericarditis etiology was determined by the patient´s history. The data were collected from medical records by referring to an operating sheet that we established at the start of the study. Epidemiological data, aetiology of CP, functional signs, data from physical examination, data from additional tests (electrocardiogram, radiography thoracic and cardiac imaging data) the time interval between symptoms and diagnosis (time elapsed between the onset of symptoms and diagnosis). Biological data were collected. We have specified the time interval between diagnosis and surgery (the time elapsed between the diagnosis and the surgical treatment). Informations about the surgical intervention were collected. Patient´s clinical and paraclinical data during in-hospital period and during follow-up were noted. Statistical study: all of the continuous variables are expressed as mean ± SD and the categorical variables as percentages. the Anova test or the Mann-Whitney test were used for the quantitative variables and the Fisher Exact test for the qualitative variables. A p value < 0.05 was considered statistically significant.

## Results

The mean age of our patients was 40.46±16.74 years (7.5-72 years). Sex ratio at 4 (20 male, 5 female). Smoking was the most frequently concomitant cardiovascular risk factors (n = 13, 52%), comorbidities are summarized in Table 1. Tuberculosis was observed in nearly the half of the cases (n = 11; 44%). Active tuberculosis contemporary with CP was found in two patients (8%). In nine patients, a history of confirmed tuberculosis was found but negativation of all tests confirming tuberculosis. Idiopathic CP in eight patients (32%). Post-cardiotomy CP occurs after valve replacement in three patients (12%), the time between surgery and the onset of signs of CP was respectively 2 years, 4 years and 5 years. Viral pericarditis in two patients (8%) and Mulibrey's nanism in one patient (4%). We have no post-radiation CP. The mean time between symptoms and diagnosis was 17.05±24.20 months (0.5-72 months). In nine patients (36%) the diagnosis was performed in the first six months after the onset of symptoms. Dyspnea was the most frequent symptom, found in 21 patients (84%) with predominantly NYHA class II (n = 10, 40%). NYHA class III or IV dyspnea was noted in seven patients (28%), all symptoms are listed in Table 2.

**Table 1:** patient´s comorbidities

Comorbidity	Number (n)	Percentage (%)
Tabagism	13	52
Ethylism	5	20
Diabetes	1	4
Systemic arterial hypertension	0	
Coronary artery disease	0	
Valvulopathy	3	12
Stroke	1	4
Chronic obstructive pulmonary disease (COPD)	1	4

**Table 2:** clinical signs

	Number of the case (n)	Percentage (%)
Dyspnea	21	84
Chest pain	6	24
Liver pain on exertion	5	20
Digestive signs	4	16
Asthenia	2	4
Palpitations	1	8
Weight loss	1	4
No symptoms	1	4
Pericardial knock	2	8
Muffled heart sounds	9	36
Jugular venous distension	18	72
Hepatojugular reflux	14	56
Hepatomegaly	19	76
Peripheral edema	16	64
Splenomegaly	2	8
Ascites	10	40
Pleural effusion	4	16

The most frequent physical sign was hepatomegaly, jugular venous distension, peripheral edema, hepatojugular reflux and ascites. Blood pressure was normal in all patients, pulsus paradoxus was not found in any patient (Table 2). Preoperative electrocardiogram was abnormal in most patients (n = 24, 96%). The most frequent electrical sign was diffuse repolarization disorders (48%, n = 12) followed by low voltage (36%, n = 9). Three patients (12%) had a right branch block. Atrial fibrillation (AF) was found in seven patients (28%) and flutter in two patients (8%). Chest x-ray revealed pericardial calcifications in eleven patients (44%), cardiomegaly in six patients (24%) and a pleural effusion in six patients (24%). Mean plasma creatinine level was 73.9±16.48 µmol/L, mean creatinine clearance was 115±17.38 ml/min (89, 5-148). Abnormal liver function tests in 13 patients (52%), hepatic cirrhosis was observed in two patients, laboratory test anomalous are listed in Table 3. On transthoracic echocardiography (TTE), biatrial dilation was the most frequent sign followed by pericardial thickening, septum notch and inferior vena cava plethora. Right ventricular (RV) dysfunction was frequent observed in 10 patients (40%), left ventricular (LV) dysfunction was noted in three patients (ejection fraction respectively 40, 40 and 32%), and Mean LV ejection fraction was 53.6±12.9%. In Doppler study E/A ratio > 0.8 was present in all patients and respiration-related variation of mitral inflow velocities was frequent present in 60% of patients, echocardiographic data are detailled in Table 4. Cardiac catheterization was performed in 21 patients (84%). A Dip- and-plateau (square root sign) and equalization of end diastolic pressures in right and left cardiac chambers were found in all patients. Pressure in different cardiac chambres are listed in Table 5. Cardiac computed tomography (CT) was performed in 12 patients (48%). The commonest abnormalities were pericardial calcification noted in seven patients, pleural effusion in six patients, and pericardial thickening in four patients. Cardiac magnetic resonance imaging (MRI) was performed in five other patients (20%). Pericardial thickening was observed in five patients and pericardial calcifications in two patients.

**Table 3:** laboratory tests

Anomaly	Number of the case (n)	Percentage (%)
Anemia	5	20
Thrombocytopenia	8	32
Leukopenia	2	8
Hepatic cytolisis	4	16
Cholestasis	9	36
Increased Acid Uric Level	1	4
Hyponatremia	4	16
Decreased Prothrombin Time	12	48

**Table 4:** echocardiographic signs

Echocardiographic signs	Number of patients	Percentage (%)
Biatrial dilation	24	96
Septal notch	20	80
Pericardial thickening	20	80
Inferior vena cava dilation	17	68
Inferior vena cava plethora	12	48
Pericardial calcifications	9	36
Pericardial effusion	6	24
Left ventricular dysfonction	3	12
Right ventricular dysfonction	10	40
Prosthetic valve and native valvulopathy	4	16
E/A>0.8	25	100
Respiration-related variation in mitral inflow velocities	15	60
Respiration-related variation in tricuspid inflow velocities	7	28
E/A>2(restrictive mitral flow)	4	16
Reflux in hepatic vein	2	8
Pulmonary regurgitation flow: Dip-plateau	6	24

E: Protodiastolic wave of mitral flow, A= End-diastolic wave in mitral inflow

**Table 5:** cardiac catheterization

Séries	Average	Extremes
Mean right atrial pressure (mmHg)	22.38±5.67	(21-31)
Mean right ventricle end-diastolic pressure (mmHg)	20.07±3.8	(15-30)
Mean diastolic pulmonary arterial pressure (mmHg)	25±8	(16-45)
Mean wedged pulmonary artery pressure (mmHg)	22.32±5	(15-31)
Mean left ventricle end diastolic pressure(mmHg)	26.5±9	(20-44)

Regarding medical treatment prior to surgery, 23 patients (92%) were on diuretics, eleven patients (44%) were on vitamin-K antagonist treatment (patients with supraventricular rhythm disturbances and with valvular heart prosthetic). Three patients (12%) had undergone one previous sternotomy for valvular replacement. Pericardiectomy was performed in all our patients within 2.9±3.19 months, it was subtotal in 96% of cases (n = 24) and partial in only one patient. The midline sternotomy was performed in all patients. Cardiopulmonary bypass (CPB) support was necessary in two patients (8%) because major bleeding for one and a tricuspid annuloplasty of De Vega for tricuspid insufficiency for the other. No other concomitant cardiac surgical procedures performed at the same time as pericardiectomy. CPB support time was respectively 33 min and 46 min. Transfusion was necessary in seven patients (28%), average transfusion requirements are five red blood cell concentrates, six fresh frozen plasma and nine platelet concentrates. The use of catecholamines was necessary in 10 patients (40%). Extubation was accomplished the first postoperative day in 17 patients (68%). Mean length of stay in the intensive care unit was 5.86±2.02 days (1-36 days). Mean time spent with a chest drain in place was 4.53±6.125 days (1-26 days). The mean length of hospital stay was 26.27±14.23 days. Anatomopathological study of pericardium was performed in 16 patients (64%). A fibrotic aspect was observed in 14 patients and an inflammatory aspect in two patients. There were no specific signs of malignancy or tuberculosis.

The most frequent postoperative complications were pleural effusion requiring surgical drainage in five patients (20%) and bleeding noted in four patients (16%) (hemopericardium in two patients, hemomediastinum in one patient, and hemothorax with respiratory distress in one patient, in these patients, reoperation was necessary) and low cardiac output observed in three patients (12%). Postoperative AF was noted in one patient (4%) (Table 6). In-hospital mortality was 12%: three early postoperative deaths because of a low cardiac output and severe RV dysfunction. Among the 22 patients who survived, 17 patients were follow-up from 14 months to 21 years (mean average follow-up = 5.67 years), we lost five patients to follow-up. Among survivors a regression of dyspnea was observed within 1.8 months in 82% of patients (n = 14). There was no death during the year after the pericardiectomy, but during follow-up, one patient (4%) died 2 years after pericardiectomy, the cause of death was worsening of a hepatic cirrhosis existing before pericardiectomy. Using univariate statistical analysis significant risk factors for global mortality (in-hospital and late mortality regrouped) included NYHA class over II (classes III and IV regrouped) (p = 0.046) and right ventricular dysfunction on transthoracic echocardiography (TTE) (p = 0.023). CPB support use was a risk factor for in-hospital (p = 0.003) and global mortality (p = 0.001). Tuberculosis as aetiology does not influence in-hospital and late mortality (Table 7).

**Table 6:** post-operative complications

	Number of patients	Pourcentage (%)
Deaths	3	12
Pleural effusion	5	20
Bleeding	4	16
Low cadiac output	3	12
Sepsis	2	8
Decompensation COPD	1	4
Bronchopulmonary infection	1	4
Atrial fibrillation	1	4
Reoperation	2	8

**Table 7:** risk factors of in-hospital and global mortality

Risk factors	In-hospital mortality	Late mortality	Global mortality
Increased cardiothoracic ratio	p = 0.430	p = 0.778	p = 0.81
Age >44 ans	p = 0.260	p = 0.667	p = 0.16
Male gender	p = 0.633	p = 0.889	p = 0.21
CP post cardiotomy	p = 0.230	p = 0.889	p = 0.54
CP post-tuberculosis	p = 0.697	p = 0.556	p = 0.42
COPD	p = 0.920	p = 0.889	p = 0.61
NYHA III-IV	p = 0.070	p = 0.045	**p = 0.046**
Hepatomegaly	p = 0.570	p = 0.778	p = 0.81
Cirrhosis	p = 0.843	p = 0.111	p = 0.27
Cholestasis	p = 0.59	p = 0.500	p = 0.37
Hyponatremia	p = 0.338	p = 0.857	p = 0.90
LV dysfunction	p = 0.230	p = 0.18	p = 0.54
**RV dysfunction**	p = 0.055	p = 0.068	**p = 0.023**
Pericardial calcifications (chest radiography, TTE, CT, MRI)	p = 0.27	p = 0.33	p = 0.06
**Cardiopulmonary bypass**	**p = 0.003**	p =0.66	**p = 0.001**
Catecholamine use	p = 0.625	p = 0.333	p = 0.87
Blood transfusion	p = 0.700	p = 0.222	p = 0.78
Platelet transfusion	p = 0.843	p = 0.111	p = 0.27

## Discussion

The age of patients with CP varies from one study to another. It ranges between 40 and 66 years [4,6-9]. In our study, disease affects young patients, same findings are reported by Lin *et al*. [9] and Ghavidel *et al*. [4] (mean age respectively at 40.1±15.5 and 46.6±14.9 years). Younger age (mean 28.8±10.44 years) was reported by Yangni-Angate *et al*. [10]. In this study the aetiology of pericarditis was tuberculosis in 99% of cases. In Western countries the average age of discovery of CP is in the fifth and sixth decades as shown by Busch *et al*. [6], Kang *et al*. [7] and Tokuda *et al*. [8]. This difference could be explained by the difference in etiologies between countries. In our study only one patient was a child aged seven years and six months (Mulibrey nanism). Yangni-Angate *et al*. [10] had also children among their patients. According to literature, CP affects mainly adults and seems rare in childhood and exceptional in infants [11,12], explained by the etiologies of CP which predominate in adults and which are uncommon in children. A male predominance is noted in our study and in the most of studies without obvious explanation [6,9]. In our study, tuberculous and idiopathic CP were the most common. Eleven patients (44%) had tuberculous CP, authors from Asian countries, Africa, Iran and South Africa have reported tuberculosis as cause of CP in 22.2% to 91% of cases. Tuberculosis is responsible of early or late pericardial constriction, which occurs between a few months and several years [10,13-18] while in western countries tuberculous CP is uncommon and main etiologies are: post-surgical and post radiation CP predominating in elderly [19].

Confirmation of tuberculosis origin of the CP is not always obvious. In the study by Mutyaba *et al*. [18], the tuberculosis was retained as the cause of constriction in 90.9% of patients but was only confirmed in 32.7% cases explained by the occurrence of tuberculous pericardial constriction much later after the active pulmonary tuberculosis infection or the ineffectiveness of the treatment of pulmonary tuberculosis[9]. According to some studies [18,20-22], the most common causes of pericardial constriction were idiopathic or viral (42-49%), post-cardiotomy (11-37%), post-radiation (9-31%), connectivitis (3-7%), post-infectious including tuberculosis (3-6%) and other uncommon causes (<10%) including neoplasia, thoracic trauma, uremic pericarditis, asbestosis, sarcoidosis and the administration of certain drugs. Constrictive pericarditis post-cardiotomy is common in Western countries, it occurs after a few months to a few years after any cardiac surgery, particularly valve replacement and coronary artery bypass grafting (CABG) [23,24]. The occurrence of constriction could be explained by post-operative hemorrhage and the use of foreign material during the procedure, which causes a chronic inflammatory reaction and the onset of fibrosis [3]. This etiology is relatively rare in our study. Constrictive pericarditis post-radiation is also a common cause of pericardial constriction in Western countries. Pericardial constriction can occur 5 to 10 years after radiotherapy and is most often associated with pancarditis [3,20,25]. None of our patients had a history of radiation therapy.

Idiopathic CP is frequent in our study and in the literature [20-22]. According to some authors, it could be explained by anterior viral pericarditis without microbiological evidence [3]. Etiology of constrictive pericarditis influences not only short-term but also long-term outcome following pericardiectomy. Idiopathic and inflammatory pathogenesis was associated with the best in-hospital and long-term survival rates, while radiation-induced constrictive pericarditis showed very poor prognosis [26]. Cardiac involvement is the most serious disease during Mulibrey nanism, characterized by pericardial constriction or myocardial hypertrophy with fibrosis [27]. Mulibrey nanism is a rare cause of CP. Only one of our patients presented a CP as part of Mulibrey nanism. Due to the scarcity of the disease and non-specific symptoms, there is often a delay in diagnosis. The time between onset of symptoms and diagnosis was long in our study and other studies, it was 19.7±6 months in the study of Nataf *et al*. [28]. In a more recent study, the diagnosis was made within six months in 57.7% of patients, between six and 12 months in 17.5% of patients and more than 12 months in 25.8% of patients [6]. Constrictive pericarditis should be suspected in any patient for whom the severity of heart failure is disproportional to the degree of myocardial dysfunction. The commonest symptom is dyspnea, literature findings are similar to ours [3,4,17]. Constrictive pericarditis can be asymptomatic, which is the case of one of our patients, a similar case was published by Sheth AA *et al*. it was a 28-year-old man in whom a CP was discovered in a context of liver dysfunction [29]. The most common physical signs described in the literature are hepatomegaly (23.4%-100%), jugular venous distension (52%-65%), ascites (8.9%-90%) peripheral edema (8.9%-84%). Unilateral or bilateral pleural effusions are often associated (35% to 79.3%) [4,9,17,18,21,30,31], these results are similar to ones we found. The paradoxical pulse of Kussmaul is an evocative physical sign, it was found in 63.6% in the series of Mutyaba *et al*. [18], 22% in the series of Lin *et al*. [9], 19% in the series of Ling *et al*. [21] and 16% in the series of Cameron *et al*. [20]. It has not been noted in our patients.

A supraventricular arrhythmia was found in 36% of our patients which is in concordance with Kumawat *et al*. [32] who found AF in 34.8% of their patients. A less AF (16%) was found by Ling *et al*. [21]. In our patients a low voltage was frequent and it is frequently found in several studies such as those of Ling *et al*. (27%), Bashi *et al*. (75%) and Mutyaba *et al*. (63.6%) [18,21,31]. Concerning chest x-ray, Cardiac calcifications were frequent (44%) in our patients which is close to the literature, Kumawat *et al*. [32] noted them in 37% of their patients and Yangni-Angate *et al*. [10] in 50% of cases. it is important to note that the presence of calcifications reflect the duration more than the cause of the disease [31]. Pleural effusion can be associated in 60% of cases [33]. Echocardiography is the most useful initial investigation. Our echocardiographic findings are consistent with those of the literature. Pericardial thickening was noted in 75% of the cases of Lin *et al*. and in 42% of the cases in of Bashi *et al*. [9,31]. According to Ling *et al*. [34], the sensitivity of transthoracic echocardiography for the detection of pericardial thickening is only 46%. This low sensitivity of detecting thickening is explained by the technical limitations of TTE as well as the inhomogenous thickening of the pericardium. The inferior vena cava plethora is an interesting sign to make the diagnosis of CP, according to some authors constriction unlikely if not dilated inferior vena cava and E/A ratio not superior to 0.8 [35]. In our study, patients without dilation of the inferior vena cava were on a high dose of diuretic. Biatrial dilation is the most common sign found in our patients, it is a very suggestive but not very sensitive sign [36]. The Doppler analysis is a main exploration time allowing not only to highlight the signs of constriction but also to differentiate CP from restrictive myocardiopathy [37]. Variation in mitral inflow velocities observed in our patients is a very sensitive sign of pericardial constriction but also not specific of CP. Velocities in mitral annulus in tissue doppler are normal during CP while decreased in restrictive myocardiopathy. Tissue Doppler allows differentiation between these two diseases with a sensitivity of 95% and a specificity of 96% for CP [38]. It was only measured in four of our patients, explained by the unavailability of this technique at the time of performing echocardiography and was greater than 7 cm/s in all cases. The dip-and-plateau present in one quarter of our patients is an evocative but not specific sign. RV dysfunction is common in our patients. The RV systolic function is significantly altered during CP [39]. Epicardial inflammation and fibrosis can directly affect myocardial contractility and lead to the formation of adhesions between the myocardium and the parietal pericardium. These adhesions can both reduce the preoperative RV systolic performance and lead to additional peroperative myocardial damage [40]. Prolonged and progressive constriction can lead to RV myocardial atrophy [41,42].

Cardiac catheterization can be used when echocardiography, and multimodality cardiac imaging provide equivocal results or when a mixed cardiac pathology requires further evaluation, it is a sensitive (100%) and specific (97%) examination for the diagnosis of CP allowing definitive confirmation of the diagnosis and formally elimination of a right or a bilateral endomyocardial fibrosis and a restrictive myocardiopathy and evaluation of the severity of coexisting valvular lesions [43,44]. Cardiac catheterization was performed in 84% of our cases, dip-and-plateau and adiaslia was found in all patients, some results are published by McCaughan *et al*. [45], in the Busch´s study dip-and-plateau was noted in 77% of cases [6]. The average pressure measured in heart chambers of our patients was consistent with those of other studies it was around 20 mmHg. The subtotal pericardiectomy was the preferred intervention performed in all our patient. Chowdhury *et al*. [46], were demonstrated that subtotal pericardiectomy was associated with lower perioperative mortality, less frequent postoperative low flow syndrome, earlier improvement of hemodynamic state allowing better long-term survival. Primary use of cardiopulmonary bypass support is not recommended due to increased diffuse bleeding during pericardial resection following systemic heparinization. Indeed, the use of cardiopulmonary bypass support should only be necessary if other coexisting cardiac lesions will be operating in same time or in the event of the occurrence of hemorrhagic lesions during the procedure which is the case for our two patients. There is no link between the type of operation (subtotal or partial pericardiectomy) and the indication for the use of cardiopulmonary bypass support [28,46,47]. In our study, the perioperative mortality rate was 12% which is in line with previous cohorts in which mortality was between 6.1% and 14% [20-22,26,46,48]. In these studies, the most frequent cause of early death was a low cardiac output [1,8], which is noted in our patients. The commonest complications observed by Biçer *et al*. [17] where pleural effusion (12.8%) followed by pulmonary infection (8.5%), in the study of Lin *et al*. [9] pleural effusion was found in 47% of cases followed by low cardiac output in 31% of cases. In other studies, acute renal failure, AF and low cardiac syndrome were the most common complications after pericardiectomy [4,8,30]. The most common post-operative rhythm and conduction disorder are AF and atrioventricular block requiring implantation of pacemaker. Post-pericardiectomy AF was noted in 8.4% of patients of Tokuda *et al*. [8] and in 4% of cases in our study. In the study by Bush *et al*. [6] Atrioventricular block was noted in 7.2% of patients, this complication was uncommon in patients published by Tokuda *et al*. (0.6%) [8], and it was not noted in our series.

Reoperation was inevitable in two cases (8%) of our series in order to evacuate a hemomediastinum for one and a hemothorax for the other. In litterature, bleeding was the causes of reopertion in 2.1% to 19.6% of cases [6,8,17]. According to several studies, the results of the pericardiectomy are satisfactory, since a significant improvement in the symptoms is observed in more than 80% of the patients [5,10,17,46]. Our results are in concordance with literature, a regression of dyspnea was observed within 1.8 months in 82% of patients. During the first post-operative year, clinical signs of right heart failure disappeared in 53% of our patients within a mean duration of 1.62 months, this was noted in 80% of the patients beyond the first year postoperative. During follow-up, no recurrence of CP was noted. The functional improvement obtained after pericardiectomy is maintained in long-term, except in certain cases. In Ling's study, signs of heart failure recurred within an average of 7.1 months in 31% of patients, despite radical surgery [21]. The reappearance of signs of right heart failure after pericardiectomy may be related to incomplete pericardial decortication, extension of pericardial calcifications to the myocardium, and myopericardial involvement by the same pathological process as radiotherapy [21,49]. Regarding the evolution of echocardiographic signs during the first post-operative year, the disappearance of constriction was noted in all patients (100%). A recent study [50] comparing the preoperative and postoperative echocardiography demonstrated a significant reduction in the congestion of the inferior vena cava from 100% to 15% of cases, a reduction in the size of the left atrium from 39.33±10.52 mm to 34.45±10.08 mm, a reduction in the paradoxical septum and respiratory variations in mitral inflow velocity. In our patients with preoperative RV dysfunction, improvement in RV function was noted in a single patient within 14 months. Improvement in the RV function after surgery occurs in a minority of patients, this could be explained by a high frequency of myocardial involvement and a possible additional injury during the intervention [40]. After a pericardiectomy, an abrupt increase in filling volume can lead to acute RV dilation and dysfunction [40]. Post-pericardiectomy RV dysfunction may also result from ischemia caused by an imbalance between supply and need for oxygen to the RV myocardium due to the sudden increase in RV wall tension by his extension after pericardiectomy.

Independent predictors of late survival were age, NYHA class, previous radiation, LV dysfunction, hepatomegaly, cirrhosis, hyponatremia, need of high doses of diuretics, rise of bilirubin´s level [9,10,18,21,22,26,49]. Previous radiotherapy was the most powerful predictor of all outcome measures [26]. In our study the use of CPB is the only predictor of in-hospital mortality this result is consistent with those found in other studies [8]. Right ventricular dysfunction on echocardiography was a predictor of global mortality, which is contradictory to the results published by Choudhry *et al*. [40] which showed that the function of RV is significantly impaired during CP but this RV dysfunction does not influence long-term results after pericardiectomy [21]. Functional class NYHA over class II is a risk factor for mortality which is in line with a lot of studies demonstrating a correlation between the functional class and operative mortality [10,26]. Tuberculosis as etiology of CP, had no impact on mortality which is in line with other studies [26]. This is the first study that relates a Tunisian experience on treating constrictive pericarditis. However, there were some limitations that warrant mention, because of the retrospective aspect of our study several clinical, and imaging data were missing from medical records and additional examination reports. Another important limitation is the small size of our sample despite a bicentric study explained by the low incidence of constrictive pericarditis resulting in restricted statistical analysis: therefore, the results of the survival analysis could not be extended by multivariate analysis of the subgroups and therewhy we regrouped patients with in-hospital and late mortality to determine risk factors of mortality. A large-scale study is to be carried out in order to establish the prognosis of the CP.

## Conclusion

The particularity of our study is predominance of post-tuberculosis CP and frequency of RV dysfunction. Treatment of CP by pericardiectomy is effective with a significant improvement of patients´ functional status and favorable outcome at short and long-term. Nevertheless, hospital mortality is not negligible and depends on many factors.

### What is known about this topic

- Constrictive pericarditis is a rare and severe disease;
- Constrictive pericarditis is difficult to diagnose, usually with delayed diagnosis;
- Pericardiectomy with complete decortication is the definitive treatment.

### What this study adds

- The first Tunisian study about constrictive pericarditis;
- Particularities of our patients: predominance of tuberculous pericarditis and frequency of right ventricular dysfunction associated with constrictive pericarditis;
- From our study we reviewed literature data concerning clinical, paraclinical characteristics, the etiologies and the evolution of constrictive pericarditis after pericardiectomy and determine risk factors of mortality and verify if the tuberculous etiology influences the postoperative mortality of patients with constrictive pericarditis.
